# Late detection of schizophrenia patient VIH-VHC

**DOI:** 10.1186/1742-4690-9-S1-P93

**Published:** 2012-05-25

**Authors:** Karine Bartolo, Nathalie Labrune, Isabelle Jaquet

**Affiliations:** 1 Chu Sainte Marguerite, Marseille, France

## Introduction

After several years, some patients on TSO, became custumers of BZD (40 tablets per day).

During the maintenance with these patients, we can notice that this consummations are rythmed by periods where there is a new outbreak of productives symptoms.

Auditory and visual hallucinations with influence syndrom, persecution syndrom, tense boarder, social isolation, peculiarity contact, mutilations to-himself, disjointed thought.

These symptoms can’t be withdrawal symptoms or a decrease of their consummation.

## Methode

### Case report

HDM: He is man, 41 years, co-infected patient (VIH and VHC).

ATCD: He’s poly-addict with BZD in first line drug.

### Symptoms

- persecution

- tense boarder

- suicidal ideas

- decrease motivation

- decrease memory

- violent acting

### Treatment and observance

He was having TSO from ten years (buprenorphine 16 mg per day) and a large number of hospitalisation before starting the psychiatric disease treatment.

When the diagnosis was established, the treatment LAAA is administrated and the patient progressively gave up the BZD consummation.

The symptoms progressively disappeared. The only time he was hospitalised is because the withdrawal of BZD was too violent.

### Diagnosis

The diagnosis of schizophrenia with deficit disorders was imput to this patient when the symptoms are stand out ,specialy , when he had no drugs: the BZD, taken in exes.

## Results

We observe that the number of hospitalisations has decreased from 2 since the begining of the new long acting atypical antipsychotics (LAAA).

## Conclusion

We must make a difference between psychiatrics symptoms and associated drugs withdrawal clinic signs when we consider poly-drug users who are partially stabilized by a TSO.

